# Hepatoprotective Screening of *Seriphidium kurramense* (Qazilb.) Y.R. Ling

**DOI:** 10.1155/2021/9026731

**Published:** 2021-12-06

**Authors:** Maroof Ali, Hidayat Hussain, Amjad Hussain, Abdur Rauf, Wahid Hussain, Manzoor Ullah, Safdar Abbas, Yahya S. Al-Awthan, Omar Bahattab, Muhammad Khan, Ahmed Olatunde, Zainab M. Almarhoon, Yahia N. Mabkhot, Mohammed M. Alshehri, Sevgi Durna Daştan, Mohamed Fawzy Ramadan, Javad Sharifi-Rad

**Affiliations:** ^1^College of Life Science, Anhui Normal University, Wuhu 241000, China; ^2^Leibniz Institute of Plant Biochemistry, Department of Bioorganic Chemistry, D-06120 Halle (Saale), Germany; ^3^Department of Chemistry, University of Okara, Okara, Pakistan; ^4^Department of Chemistry, University of Swabi, Anbar KPK, Pakistan; ^5^Department of Botany, Government Post Graduate College Parachinar, Kurram 26000, Pakistan; ^6^Department of Botany, University of Science & Technology Bannu, Pakistan; ^7^Department of Biochemistry, Faculty of Biological Sciences, Quaid-i-Azam University, Islamabad, Pakistan; ^8^Department of Biology, Faculty of Science, University of Tabuk, Tabuk, Saudi Arabia; ^9^Department of Biology, Faculty of Science, Ibb University, Ibb, Yemen; ^10^Department of Biochemistry, Abubakar Tafawa Balewa University, Bauchi 740272, Nigeria; ^11^Department of Chemistry, College of Science, King Saud University, P.O. Box 2455, Riyadh 11451, Saudi Arabia; ^12^Department of Pharmaceutical Chemistry, College of Pharmacy, King Khalid University, Abha, Saudi Arabia; ^13^Department of Pharmaceutical Care, Ministry of National Guard-Health Affairs, Riyadh, Saudi Arabia; ^14^Department of Biology, Faculty of Science, Sivas Cumhuriyet University, 58140 Sivas, Turkey; ^15^Beekeeping Development Application and Research Center, Sivas Cumhuriyet University, 58140 Sivas, Turkey; ^16^Deanship of Scientific Research, Umm Al-Qura University, Makkah, Saudi Arabia; ^17^Department of Agricultural Biochemistry, Faculty of Agriculture, Zagazig University, Zagazig 44519, Egypt; ^18^Facultad de Medicina, Universidad del Azuay, Cuenca, Ecuador

## Abstract

Investigation on medicinal plants' therapeutic potential has gained substantial importance in the discovery of novel effective and safe therapeutic agents. The present study is aimed at investigating the hepatoprotective potential of *Seriphidium kurramense* methanolic extract (SKM) against carbon tetrachloride- (CCl_4_-) induced hepatotoxicity in rats. *S. kurramense* is one of the most imperative plants for its various pharmacological activities. Therefore, this study was aimed at evaluating the hepatoprotective potential against CCl_4_-induced liver toxicity. The serum samples were analyzed for alanine aminotransferase (ALT) and aspartate aminotransferase (AST) together with the oxidative stress mediator levels as nitric oxide (NO), malondialdehyde (MDA), glutathione (GSH), reduced glutathione (GSH), and superoxide dismutase (SOD) as well as peroxidation and H_2_O_2_ activity. CCl_4_ administration resulted in an elevated free radical generation, altered liver marker (AST and ALT) enzymes, reduced antioxidant enzyme, and increased DNA damage. Methanolic extract of *S. kurramense* decreased CCl_4_-induced hepatotoxicity by increasing the antioxidant status and reducing H_2_O_2_ and nitrate content generation as well as reducing DNA damage. Additionally, SKM reversed the morphological alterations induced by CCl_4_ in the SKM-treated groups. These results demonstrated that SKM displayed hepatoprotective activity against CCl_4_-induced hepatic damage in experimental rats.

## 1. Introduction

*Seriphidium kurramense* (Qazilb) Y.R. Ling is an important medicinal and economic plant of the family Asteraceae and endemic to the tribal district, Upper Kurram, Pakistan, and also transported to different parts of the country for the extraction of new drugs [1]. By surveying the literature, it has been confirmed that several medicinal plants of Pakistan collected from Khyber Pakhtunkhwa, including tribal districts, have been screened out. The earlier studies showed that *S. kurramense* was revealed to have the highest medicinal level and also used for insecticide purposes [1, 2]; Kafeel et al., 2018; [2], by clarifying the mechanism underlying the phytochemicals and biological activities of *S. kurramense* and providing a reference to address protective potential. Carbon tetrachloride (CCl_4_) is a well-renowned industrial solvent known to cause hepatotoxicity. Free radicals derived from CCl_4_ are involved in covalent binding to the macromolecules, which also cause lipid peroxidation [3, 4]. It has also been used as a dry-cleaning agent in industries, as a catalyst in polymer reactions, as a solvent in cleaning metals, and as granule fumigant [5, 6]. Numerous studies have been conducted on CCl_4_, which showed toxicity in various pathophysiological conditions [7]. Different studies have shown that CCl_4_ intoxication is constrained to the liver and causes oxidative damage to the tissues of the lung, kidney, heart, brain, testis, and blood [8, 9]. Due to succeeding CCl_4_ exposure, lipid peroxide and protein carbonyl levels were identified in tissues isolated from the lung, kidney, and testis of rats [10]. CCl_4_ requires cytochrome P450 (phase I system) for the activation of metabolic system in the liver to initiate reactive radical species such as proxy trichloromethyl (^·^OOCCl_3_) and trichloro-4 methyl (^·^CCl_3_), which are involved in increasing lipid peroxidation and protein oxidation causing liver damage [11]. Both radicals are further involved in the initiation of alkoxy (RO^·^) and peroxy (ROO^·^) radicals through their action on the polyunsaturated fatty acids [12]. The silymarin extract obtained from the *Silybum marianum* is a mixture of polyphenols and flavonoids. Commercially prepared silymarin consists of various flavonoids, such as silidianin and silichristin, isosilibinin (isosilybin A and B), and silibinin (silybin A and B). Silibinin is the key component of this mixture. Silymarin has been found to show antioxidant potential and stabilize the membrane; it also inhibits fibrogenesis, reduces inflammatory reaction, and provokes hepatocyte regeneration. These results were verified through several clinical trials [13–16]. Therefore, the present study was designed with the aim to evaluate their biological activities including hepatoprotective potential of *Seriphidium kurramense* methanol extract (SKM) against CCl_4_-induced hepatotoxicity in rats. Our results showed that SKM treatment significantly abolished CCl4-induced hepatotoxicity via attenuating free radical generation, boosting antioxidants, and preventing DNA damage.

## 2. Results

### 2.1. Effects of SKM on Liver Biomarker Indices

The ALT and AST activities and globulin levels were significantly elevated, while the level of albumin and total protein was decreased in the CCl_4_-treated rats compared to the control group (Table 1). However, the SKM administration reverses CCl_4_ effects by reducing ALT and AST activities and the altered level of albumin, globulin, and total protein. Notably, the SKM+CCl_4_-treated groups showed results comparable to the group treated with silymarin. Interestingly, SKM effects on the liver biomarkers (ALT, AST, albumin, globulin, and total protein) were recorded to be dose-dependent (Table 1).

### 2.2. The Effects of SKM on the Activities of CAT, POD, and SOD and the Level of GSH

Table 2 shows the effect of SKM on the activities of antioxidant parameters in experimental animals subjected to CCl_4_ toxicity. The antioxidant enzyme (CAT, POD, and SOD) activities and the level of GSH were significantly increased in the CCl_4_-treated rats administered with SKM compared to rats treated with CCl4 only. The administration of SKM to CCl_4_-treated rats showed a dose-dependent increase in the activities of CAT, POD, and SOD and the level of GSH. Consistently, the increased antioxidant indices (CAT, POD, SOD, and GSH) were greater in the group treated with SKM only compared to the groups treated with both SKM+CCl_4_ as well as the control groups (normal control and silymarin group).

### 2.3. The Effect of SKM on the Level of TBARS, Nitrite, and H_2_O_2_

The level of TBARs, nitrite, and H_2_O_2_ was recorded to be significantly elevated in the group treated with CCl_4_ only compared to the groups treated with the SKM and silymarin as well as the control group (Table 3). The elevation recorded of these markers of oxidative stress in the groups treated with CCl_4_ only was reduced in a dose-dependent manner after SKM administration. The results obtained in the CCl_4_ groups administered with SKM were comparable to the CCl_4_ group treated with silymarin.

### 2.4. SKM Treatment Attenuated CCl_4_-Induced DNA Injury

**Figure 1:**
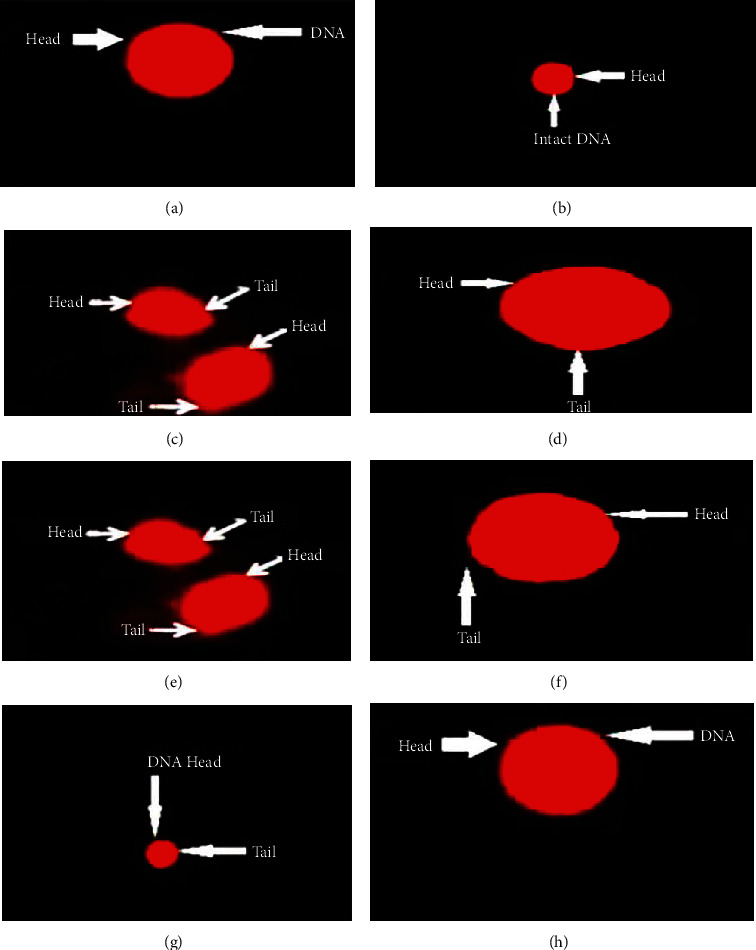
The fluorescence photomicrograph of *Seriphidium kurramense* methanol extract effect on DNA of hepatic cells: (a) control group, (b) vehicle control, (c) CCl_4_ only, (d) CCl_4_+rutin, (e) CCl_4_+low dose (150 mg/kg), (f) CCl_4_+high dose (300 mg/kg), (g) low dose alone, and (h) high dose alone.

The comet and tail length, DNA in the tail, and tail moments were significantly high, while the head length and DNA in the head were significantly low in CCl_4_-treated rats than in the control group (Figure 1, Table 4). Nevertheless, the administration of SKM to the groups treated with CCl_4_ ameliorated the DNA damage and was comparable with the group treated with both CCl_4_ and silymarin. Interestingly, the group treated with solo SKM showed better results compared to the groups treated with both CC_l4_ and SKM (Figure 1, Table 4).

### 2.5. Defensive Effect of SKM on Histoarchitecture of the Liver

**Figure 2:**
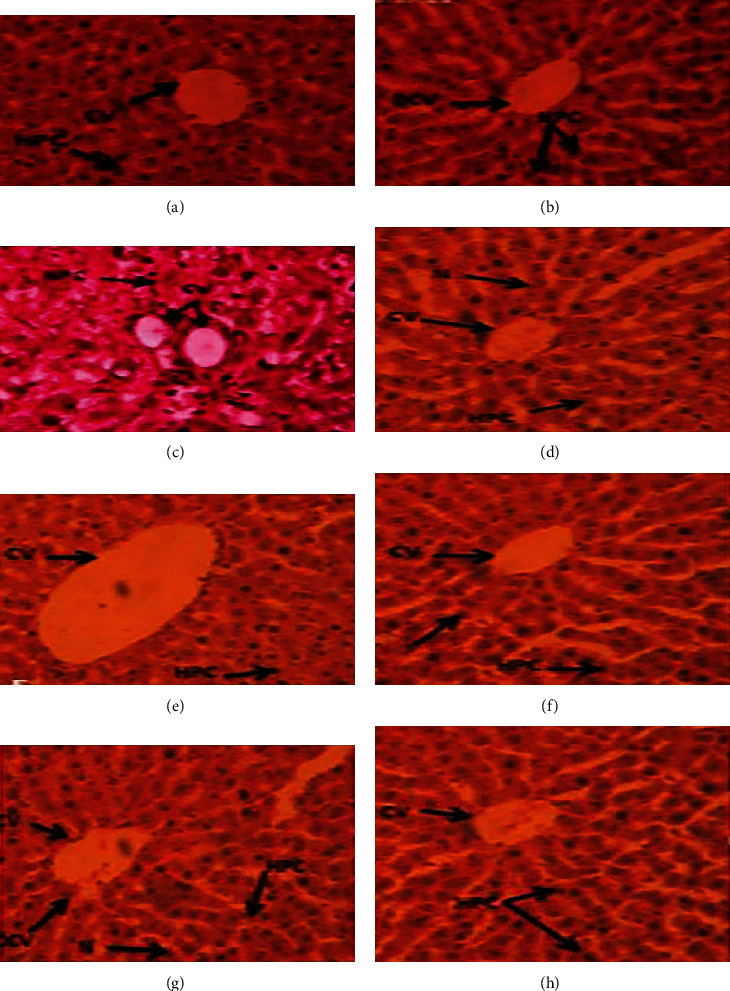
Liver histopathological observations of control and treated groups at 40x: (a) control, (b) vehicle control, (c) CCl_4_ only, (d) CCl_4_+silymarin 200 mg/kg, (e) CCl_4_+SKM 150 mg/kg, (f) CCl_4_+SMM 300 mg/kg, (g) SKM 150 mg/kg, and (h) SKM 300 mg/kg.

Hematoxylin and eosin are used to stain the thinly sliced sections of liver tissue which were microscopically photographed at 40x to examine various morphological alterations, as shown in Figure 2. Normal morphology is shown in the control group with the distinctive central vein, Kupffer cells, hepatocytes, and sinusoids (Figure 2). Furthermore, CCl_4_ treatment caused a noticeable elevation in fatty changes, cellular hypertrophy, inflammatory cell infiltrations, ballooning, and dilation of the central vein in the liver tissues. However, SKM administration (150 mg/kg) presented the hepatic structure with little fatty changes, dilation of the blood vessel, and uniform morphology of hepatocytes similar to the control group. Similarly, silymarin (50 mg/kg) attenuated the cellular alterations and distractions as expressed. The hepatic histology illustrated that SKM was a higher defensive dose (300 mg/kg) of SKM.

## 3. Discussion

Carbon tetrachloride (CCl_4_) is a well-known lethal hepatotoxin, and free radical production causes different disorders [17–19]. For centuries, experimental models have been used to investigate mechanisms of oxidant/free radical toxicity in induced chronic disorders [20]. The current study was conceded out to evaluate the ameliorative potential of *S. kurramense* against liver damage in CCl_4_-treated experimental rats. Our result corroborates with Singh et al. [21], who established the antioxidative properties of *Solanum xanthocarpum* fruit extract against drug-induced toxicity.

CCl_4_ induces oxidative stress by free radical generation, causes tissue injury by DNA damage, distressed enzymatic level, and elevated lipid peroxidation. Previously, it has been reported that CCl_4_ (1 mL/kg) administration for 4 weeks at alternating days caused hepatic fibrogenesis, injured the functional reliability of cell membrane and hepatic mitochondrial function, and increased serum enzymes and endogenous antioxidant enzyme pool [22, 23]. Recent studies reveal that CC4-treated rats showed a high intensity of liver markers ALT, AST, and ALP in the serum due to damaged hepatocellular membrane reliability [12, 24]. In the present study, the plant's methanol extract showed its protective aptitude according to its dose concentration. Moreover, the low dose (150 mg/kg) was protective, while the protective effect of the high dose (300 mg/kg) was significantly incoherent with silymarin. Our results are in accordance with the study of Sahreen et al. [12], in which they documented that the antifibrotic effects are caused by the antioxidant activity of *Rumex hastatus* D. On the other hand, serum activities of AST, ALT, and ALP are related to hepatocyte membrane damage, leaked out into plasma due to the damaged membrane. Singh et al. [25] suggested that the above serum markers' high levels can be the consequence of massive centrilobular necrosis and cellular infiltration in the liver.

Nevin and Vijayammal [8] proposed that compounds and chemicals' toxicological nature can be regularly tested by total protein estimation. Consistent with previous findings herein, CCl_4_ intoxication reduced protein levels both in the serum and at the tissue level, and SKM treatment reversed these CCl_4_-induced effects. Furthermore, a high dose of SKM showed more promising and protective effects than a low dose against CCl_4_-induced protein level dysregulation, suggesting a dose-dependent action of SKM [26]. These results showed that *S. kurramense* is a worthy candidate to inhibit the DNA damage in renal tissues. However, the antioxidant resistance system includes the enzymatic antioxidants (CAT, POD, SOD, GST, and GSH), which contributed a fundamental role in the protective system *via* scavenging free radicals. It has been reported that CCl_4_ treatment can cause a significant reduction in the CAT, POD, SOD, GST, and GSH levels [27, 28]. Interestingly, the level of GSH reduced because of its more utilization by the hepatocytes in hunting toxic radicals produced by CCl_4_. [29] also documented the decline in levels of all enzymes and GSH content in liver tissue by CCl_4_ supervision. However, SKM treatment enhanced antioxidant enzymes, including CAT, SOD, POD, GST, and GSH level, and decreased under CCl_4_-induced stress condition [29] using *Sonchus asper* as a medicinal plant. Furthermore, CCl_4_ was also showing its toxic belongings by changing the levels of TBARS, H_2_O_2_, tissue protein, and nitrite contents. Peroxidation of lipids provoked overexpression of genetic fibrogenic cytokines by motivating the collagen amalgamation and activating hepatic [30]. Herein, in agreement with previous findings: our results demonstrated reduced protein level and enhanced TBARs, H_2_O_2_, and nitrite content upon CCl_4_ administration. However, SKM treatment significantly recovered CCl_4_-induced toxicity by reducing TBARs level, H_2_O_2_ concentration, and nitrite content, likewise the silymarin-treated group. Furthermore, the high-dose treatment of SKM was more effective than the low dose.

Liver regeneration has a significant role in the resistance against chemical-induced damage [31], and its histopathological analysis is the shortest way of evaluating the toxic effect of a drug such as CCl_4_ and extract of different plants. In our results, liver histological analysis showed a high degree of liver cell damage, fibrosis, necrosis, cellular hypertrophy, and central lobule disruption in the CCl_4_-treated group compared to the normal subjects; a comparable result has been demonstrated previously by Chen et al. [32]. CCl_4_-caused oxidative damage to DNA in the mammalian cells has been pragmatic [33]. The single-stranded or double-stranded break in the DNA is because of free radicals, injuring DNA integrity [34, 35]. Our single-cell gel electrophoresis results showed the extent of DNA damage in the CCl_4_-treated group compared to the normal subject [36].

Furthermore, an increase in comet tail moment and decline in DNA percentage were observed in CCl_4_-treated rats compared to the control group. Akram et al. [37] used comet assay to report DNA damage in the rats' ovaries upon sodium arsenate treatment. Sodium arsenate treatment concentration depends on increased oxidative stress in the tissues, leading to abnormal oocytes with damaged DNA. And also, it is recommended to study the advanced techniques using SEM analysis for future study [38, 39]. However, in the present investigation, SKM treatment showed an increase in comet head length, DNA percentage, and reduction of comet length, tail length, and moment, as well as DNA percentage in the tail of the comet, suggesting its protecting effects against CCl4-induced cellular toxicity by preventing DNA damage. Notably, a high dose of SKM was more prominent and significant than a low dose.

## 4. Materials and Methods

### 4.1. Plant Material and Extraction

**Figure 3:**
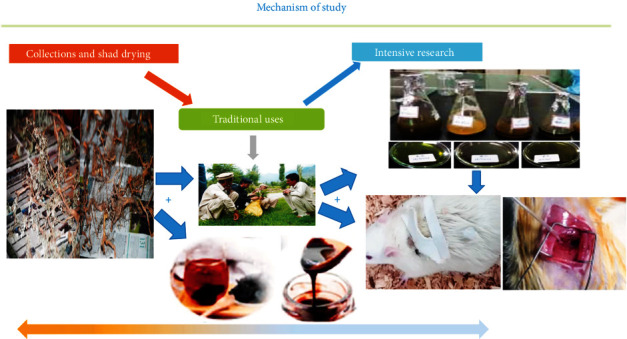
Mechanism of Seriphidium kurramense from traditional medicine to in vivo analysis.

*Seriphidium kurramense* was collected during the spring season in 2018 from Parachinar tribal district, Khyber Pakhtunkhwa (KPK), Pakistan. Dr. Mushtaq Ahmad, Professor at Quaid-i-Azam University Islamabad, identified the plant specimen. Aerial parts of *S. kurramense* were dried under shade and ground to fine powder, and about 2 kg was pulverized using an electric blender. The finely powdered sample was macerated in 4 L methanol for two weeks at room temperature. Extract of the plant was completed twice to get their soluble portions using resultant filtrate. Subsequent to obtaining cured methanol extract, particular fraction was ended by the rotary evaporator [40, 41]. Afterward, the plant extract was dried out and kept at 4°C for further explorations, while for *in vivo* analysis, CCl_4_ was preferred to rouse toxicity in the liver of *Sprague Dawley* rats as an animal model and to scrutinize them at molecular, biochemical, and tissue level. The mixture was evaporated to dryness; the slurry extract was dried completely at -70°C. The mechanism is shown in Figure 3.

### 4.2. Preparation of CCl_4_ and SKM

CCl_4_ was prepared using olive oil as a vehicle. CCl_4_ was added to the olive oil in the ratio of 30 : 70 *w*/*w*. The intraperitoneal injection of CCl_4_ mixed with olive oil was carried out for four weeks. Simultaneously, the plant methanol extract was administered at a dose of 150 and 300 mg/kg body weight. Silymarin was used as the reference drug at a dose of 100 mg/kg body weight. The various doses of SKM were prepared in DMSO as the vehicle for the plant extract.

### 4.3. Experiment

Fifty-six rats with 150-200 g weight were kept at the primate facility in Quaid-i-Azam University, Islamabad, Pakistan. The experimental animals were subjected to 12 h dark and light cycle, fed on standard rat feeds, and provided with water *ad libitum*. The methods for animal handling were following the institutional ethical committee's guidelines on scientific research. According to Table 5, the experimental animals were divided into eight groups (each *n* = 6). Group I served as a control, group II contained rats that were treated with DMSO (10%, 1 mL/kg), group III contained rats treated with CCl_4_ (i.p) (1 mL/kg), group IV received CCl_4_+silymarin (100 mg/mL/kg) (as a reference group), group V rats received CCl_4_+SKM (150 mg/kg, orally), group VI received CCl_4_+SKM (300 mg/kg, orally), group VII received SKM (150 mg/kg orally), and group VIII received SKM (300 mg/kg orally).

### 4.4. Collection of Blood Sample and Isolation of Organs

The experimental rats were subjected to chloroform anesthesia. The jugular vein of the unconscious animal was cut using a sharp disserting blade. The blood samples were collected into clean plain sample bottles. The blood samples were centrifuged for 15 min at 10,000 rpm. After centrifugation, the serum samples were collected and stored in appropriate sample bottles. The animals were dissected, and part of the liver organ was collected, cleaned, and stored in liquid nitrogen at 70°C for analysis of tissue homogenates. Following homogenization, the liver homogenate was centrifuged, and the supernatant was collected and was preserved in phosphate buffer formalin 10%, for histopathological observations.

### 4.5. Biochemical Analysis

ALT (alanine aminotransferase estimation), AST (aspartate aminotransferase estimation), ALP (alkaline phosphatase estimation), and albumin were estimated using diagnostic tools.

#### 4.5.1. Protein Estimation

The full soluble hepatic proteins were anticipated by the technique of Lowry et al. [42]. An amount of 80 mg of tissue was weighed of every organ and homogenized within the phosphate buffer. The organ tissues were centrifuged after homogenization at 10,000 rpm at 4°C for 20 min. The 0.1 mL sample is mixed in 1 mL basic solution and incubated for 10 min; then, Folin-Ciocalteu reagent was added to each tube with the ratio of 1 : 1 (*v*/*v*) and vortexed. The absorbance was recorded at 595 nm after 30 min incubation. BSA standard curve was used to determine soluble protein concentration.

#### 4.5.2. Globulin Estimation

Globulin evaluation was carried out using the following formula:total protein − albumin.

#### 4.5.3. Assessment of Antioxidative Profile

*(1) Catalase Assay (CAT)*. Catalase action was intended by Chance and Maehly's [42] technique with some changes. For the catalase evaluation, the reaction fusion restricted 2500 *μ*L of 50 mM phosphate buffer (pH 7.2), hydrogen peroxide 420 *μ*L (5.9 mM), and extract of enzyme (100 *μ*L). After 1 min, absorbance alteration of the reaction mixture of the solution was recorded at 240 nm. One entity, catalase activity, was distinct, since at 0.01 units per minute, there is an absorbance change.

*(2) Peroxidase Assay (POD)*. POD assay was resolved by Kakkar et al.'s [4] technique. The reactants of peroxidase assay were 1000 *μ*L enzyme extract, 100 *μ*L guaiacol (20 mM), 300 *μ*L H_2_O_2_ (40 mM), and 2500 *μ*L 50 mM phosphate buffer (pH 6.8). The absorbance change of the reaction solution was measured after 1 min at 470 nm. A change of absorbance of 0.01 units per minute's peroxidase activity was observed.

*(3) Superoxide Dismutase Assay (SOD)*. SOD assay was performed using Spitz and Larry's [43] technique. This process was initiated by using 100 *μ*L reaction mixture of 186 *μ*M PNS (phenazine methosulphate), 1200 *μ*L sodium pyrophosphate (0.052 mM; pH =7.0), and supernatant 300 *μ*L derived from the homogenate of the liver, which was integrated into the reaction mixture. The entire mixture was subjected to centrifugation for 10 min at 1500 rpm and after that at 10000 rpm for 15 min. The enzyme's reaction started with the addition of 0.2 mL of 780 *μ*M NADH and followed by the addition of 1000 *μ*L glacial acetic acid. The intensity of color estimated the amount of chromogen produced at an absorbance of 560 nm, and the results were recorded in units per milligram.

*(4) Glutathione S-Transferase Assay (GST)*. To evaluate GST, Habig et al.'s [44] method was used. The summary of all the reactants for this test includes 200 *μ*L of reduced glutathione of 1 mM, 1475 *μ*L phosphate buffer of 0.1 M (pH = 6.5), and enzyme extract of 300 *μ*L, in a total volume of 2000 *μ*L. At the absorbance of 340 nm, a change was observed. The activity of enzymes was assessed throughout with the formation of *M* conjugate per minute. The extermination molarity, 9.6 × 103 M^−1^ cm^−1^, was used as a coefficient here.

*(5) Reduced Glutathione Assay (GSH)*. The assay was performed using Jollow et al.'s [45] protocol. The homogeneous mixture of the sample contained 1000 *μ*L of 4% sulfosalicylic acid. The appetizer was kept at 4°C for 1 h and centrifuged for the next 20 min at 4°C (1200 g). The entire 3000 *μ*L volume of the reactant mixture included 100 *μ*L clean aliquot, 200 *μ*L DTNB (100 mM), and 2700 *μ*L phosphate buffer (0.1 M; pH = 7.4). The color (yellow) variation at various combinations of the reactants was calculated instantly by maintaining 412 nm of the absorbance. Reduced glutathione action was articulated as micromolar GSH per gram tissue piece.

*(6) Lipid Peroxidation (TBARS)*. This original assay was conducted using Iqbal and Wright's [46] method. The whole 1000 *μ*L of the main reacting sample included 0.1 M phosphate buffer (580 *μ*L) maintained at pH 7.4, homogenate sample (200 *μ*L), the 100 mM of ascorbic acid (200 *μ*L), and 100 mM ferric chloride (20 *μ*L). The whole reacting sample was kept at 37°C for 1 h in the incubator. The 1000 *μ*L of 10% trichloroacetic acid was added to block the reaction. The sample tubes were inserted in hot water maintained at 100°C, followed by adding 0.67% thiobarbituric (1000 *μ*L) for 20 min, and next positioned on the compressed ice-bath, and then, centrifugation for 10 min (2500 × g) was started. The amount of TBARS (lipid peroxidation) in every sample was premeditated by captivating the absorbance of supernatant against a blank reagent on a spectrophotometer at 535 nm. The outcomes were uttered using 1.56 × 105 M^−1^ cm^−1^ molar destruction coefficient for TBARS per minute per milligram tissues at 37°C.

*(7) Hydrogen Peroxide Assay (H_2_O_2_)*. H_2_O_2_ activity was executed following the Pick and Keisari's [47] protocol through umpired horseradish peroxidase-reliant oxidation of phenol red. The main constituents included 0.5 M phosphate buffer (pH = 7.0), 8.5 units of horseradish peroxidase, 5.5 nM dextrose, and 2 mL of a homogenate of tissue (hanging in 1000 *μ*L of a solution of 0.28 nM phenol red), and the whole mixture of the sample was incubated for 60 min at 37°C followed by addition of 10 *μ*L of 1 N sodium hydroxide to cease further reaction. The centrifugation was performed at 800 × g for 5 min. At the wavelength of 610 nm, the supernatant's absorbance against the blank reagent was calculated. The nM H_2_O_2_ was fed in milligram per minute per tissue using standard curve H_2_O_2_ oxidized phenol red.

*(8) Nitrite Assay*. Grisham et al. [48] experimented to authenticate the nitrite assess. The Griess reagent was used in this experiment. The sample (100 mg tissue homogenate) was deproteinized with 5% ZnSO_4_ and 0.3 M NaOH (100 *μ*L). The entire mixture was subjected to 15-20 min of centrifugation at 6400 × g. A volume of 1 mL Griess reagent along with 20 *μ*L supernatant was added in the cuvette. The absorbance of the reaction mixture was recorded at 540 nm. The curve of NaNO_2_ was used for evaluating the quantity of nitrite in samples.

*(9) Histopathological Study of Tissues*. The paraffin-rooted bruising route finished histopathology of tissue appraisal. The different steps demand obsession with a biological taster in sticky stuff, safeguarding their morphology, and avert the tissue decomposes. Consequently, the new hepatic tissues were segmented into tiny sections and set in 10% formalin. The preset tissues were more soaked, and progression in the way of a mounting succession of 50%, 70%, 90%, and 100% alcohol, heading for the tissues, maintains on a rigid solid medium and, consequently, assists slight segments to be incised. The tissues were safe on tough solid wedges, using paraffin-implanting. Slides were fixed by slicing skinny strata of the fixed-tissue tasters 3-4 *μ*m through staining with eosin and hematoxylin. Finally, the examination of slides was carried out in the light microscope (DIALUX 20 EB) at 10x and 40x and pictures using an HDCE-50B camera were taken.

*(10) Comet Assay*. To determine the DNA damage, the protocol of Dhawan et al. [29] with some modifications was used.

*(11) Lysing Solution*. The fusion of solution was made by adding 1.46 g of 2.5 M NaOH, 1.2 g of 100 mM Trizma base, and 37.2 g of 100 mM EDTA into 700 mL distilled water. By the addition of HCl or NaOH, pH was adjusted to 10. Adjustment of entire volume was made equal to 890 mL through distilled H_2_O. DMSO and Triton X increase the volume up to the required level, and then, a solution was placed on standby at room temperature. Later on, 10% DMSO and 1% Triton X were added to result in the lysing solution's final state. The whole solution was kept in the refrigerator for 30 min.

*(12) PBS Buffer (Mg^2+^, Ca^2+^)*. It was diluted by 990 mL distilled water, and the final volume reached up to 1000 mL at room temperature and regulated at pH 7.4.

*(13) Electrophoresis Buffer*. Intended for 1X buffer groundwork in distilled H_2_O, 30 mL NaOH was added to 0.5 mL of 200 mM EDTA, and the whole volume was taken up to 1000 mL. At room temperature, pH was fixed at less than 1.

*Neutralization buffer*: 48.5 g of 0.4 M Tris was mixed with 1 L of distilled water, and conc. HCl was added at room temperature to fixed pH of 7.5.

*Staining solution*: 30 *μ*g/mL stock solution of 10X ethidium bromide. Ten milligrams of ethidium bromide was added to 50 mL dist. H_2_O to obtain ethidium bromide solution. This solution was used as a staining agent. A.0.5% of low melting point agarose (LMPA) was all set through the amalgamation of 250 mg of low melting point agarose in 1% PBS solution (50 mL). Refrigeration was stopped, and temperature was stabilized at 37°C by putting in water bath earlier to use a 1% normal melting agarose (NMA). This solution resulted by adding 500 mg of NMA in 50 mL H_2_O. Suspension of the gel is formed by heating MA in 50 mL water.

### 4.6. Preparation of Slides

The NMA and LMPA solutions were all set for the slide preparation, as illustrated earlier. Slides were set *via* sinking in methanol and burn up over a fire to get rid of dirt. The small portion of the slide was sunk in warm NMA agarose and subsequently took away gradually. Dirt-free inferior surface was kept in a dish designed for solidification. The slides were dried in the air, tagged, and placed at ordinary temperature. For the extraction purpose, a minute portion of tissue was placed in a cold solution of HBSS (1 mL) containing DMSO and EDTA in a ratio of 10 : 20. Small pieces of tissues were crushed, followed by the addition of 75 *μ*L LMPA in it. On stored slides, this blend was coated and enclosed by a coverslip. Slides were placed in ice packs for the solidification of gel. After 5-10 min, gradually remove the coverslips and third coat by addition of 80 *μ*L of LMPA on the same slide; using the ice packs, they were made dried. This was further followed by removing the coverslips and putting slides in lysing solution. Sides were protected from the light and chilled for 2 h.

### 4.7. Electrophoresis

After staying for 2 h in lysing solution, the slides were removed adjusted with a flat gel kit. The newly equipped buffer was dispensed in a gel container, and every slide was placed in the buffer for 20 mins to unzip DNA. At 24 volts, the electric supply was switched on for 30 min. Slides were gradually taken away, and next is the addition of neutralizing buffer. A similar practice was repeated again and again. A 80 *μ*L of 1X ethidium bromide staining was done, and slides were covered with coverslips.

### 4.8. Slide Visualization

DNA damage was recognized through a fluorescent microscope at 40x. The degree of DNA injury was determined through the software of image investigation CASP 1.2.3. At the same time, approximating the proportion of transferred DNA and also emigrational span was done. Usually, in all tasters, 50 to100 cells were studied. To analyze the relationship between the quantities of per cell migration, numbers of cells with improved migration, feasibility, and emigrational aptitude among injured cells were counted.

## 5. Conclusion for Future Biology

*Seriphidium kurramense* possessed potent potential against CCl_4_-induced hepatotoxicity by reducing oxidative and nitrosative stress, boosting antioxidant capacities, and reducing cellular toxicity attenuating DNA damage. Moreover, the inspected plant might be measured as an impressive natural cause to widen novel drugs and present a feasible significance of treating various diseases in the mounting world.

## Figures and Tables

**Table 1:** Effect of *S. kurramense* on liver biomarkers.

Treatment	ALT (mg/dL)	AST (mg/dL)	Albumin (mg/dL)	Globulin (mg/dL)	Tissue protein (*μ*g/mg tissue)
Control (normal)	38 ± 2.16^e^	42 ± 2.16^d^	4.47 ± 0.59^a^	3.69 ± 0.38^b^	3.26 ± 0.23^a^
DMSO+olive oil	37 ± 2.16^e^	43 ± 2.16^d^	4.33 ± 0.81^a^	3.69 ± 0.42^b^	3.25 ± 0.32^a^
CCl_4_ (1 mL/kg)	109 ± 4.19^a^	106 ± 4.11^a^	1.95 ± 0.29^d^	4.07 ± 0.36^a^	1.37 ± 0.16^c^
CCl_4_+silymarin	96 ± 3.36^b^	86 ± 4.61^b^	3.07 ± 0.37^b^	3.94 ± 0.54^b^	2.67 ± 0.33^b^
CCl_4_+SKM (150 mg/mg)	86 ± 4.42^c^	84 ± 4.42^b^	2.46 ± 0.14^c^	3.17 ± 0.66^b^	1.62 ± 0.42^c^
CCl_4_+SKM (300 mg/kg)	72 ± 3.49^d^	58 ± 3.49^c^	3.14 ± 0.21^b^	3.89 ± 0.19^b^	2.75 ± 0.45^b^
SKM (150 mg/kg)	38 ± 2.16^e^	44 ± 2.16^d^	4.14 ± 0.44^a^	3.59 ± 0.31^b^	3.41 ± 0.42^a^
SKM (300 mg/kg)	39 ± 2.16^e^	42 ± 2.16^d^	4.22 ± 0.50^a^	3.62 ± 0.46^b^	3.31 ± 0.32^a^

Values are expressed as mean ± SD (7). Values with different alphabet letters down the column indicate a significant difference (*p* < 0.05). SKM: *Seriphidium Kurramense* methanol extract.

**Table 2:** Effect of *S. Kurramense* on antioxidant parameters.

Treatment	CAT (U/min)	POD (U/min)	SOD (U/mg protein)	GSH (*μ*M/g tissue)
Control (normal)	7.3 ± 0.82^a^	9.38 ± 1.2^a^	5.33 ± 0.75^a^	22.46 ± 1.32^a^
DMSO+olive oil	7.2 ± 0.78^a^	9.32 ± 1.17^a^	5.23 ± 0.86^a^	22.41 ± 1.36^a^
CCl_4_ (1 mL/kg)	2.36 ± 0.31^d^	3.01 ± 0.33^d^	2.03 ± 0.42^d^	6.42 ± 0.72^d^
CCl_4_+silymarin	5.4 ± 0.56^b^	8.1 ± 1.12^b^	4.12 ± 0.96^b^	18.42 ± 2.16^b^
CCl_4_+SKM (150 mg/mg)	4.6 ± 0.68^c^	6.23 ± 0.85^c^	3.78 ± 0.42^c^	14.56 ± 1.11^c^
CCl_4_+SKM (300 mg/kg)	5.9 ± 0.65^b^	8.52 ± 1.23^b^	4.36 ± 0.76^b^	17.23 ± 1.26^b^
SKM (150 mg/kg)	7.3 ± 0.72^a^	9.28 ± 1.09^a^	5.28 ± 0.62^a^	22.32 ± 1.06^a^
SKM (300 mg/kg)	7.1 ± 0.93^a^	9.10 ± 1.11^a^	5.24 ± 0.72^a^	22.42 ± 1.42^a^

SKM: *Seriphidium kurramense* methanol extract. Values expressed as mean ± SD (7). Means with different alphabet letters within the column indicate a significant difference (*p* < 0.05).

**Table 3:** Effect of SKM on TBARS, nitrite, and H_2_O_2_ in CCl_4_-treated rats.

Treatment	TBARS (nM/min/mg protein)	Nitrite (*μ*M/mL)	H_2_O_2_ (*μ*M/mL)
Control (normal)	24.56 ± 2.16^a^	49.24 ± 2.04^d^	0.39 ± 0.09^e^
DMSO+olive oil	24.47 ± 2.32^a^	49.23 ± 2.25^d^	0.38 ± 0.08^e^
CCl_4_ (1 mL/kg)	47.22 ± 3.17^b^	87.68 ± 4.22^a^	1.03 ± 0.26^a^
CCl_4_+silymarin	28.23 ± 1.42^c^	56.23 ± 2.17^c^	0.52 ± 0.11^c^
CCl_4_+SKM (150 mg/mg)	30.26 ± 2.42^c^	63.01 ± 2.23^b^	0.75 ± 0.15^b^
CCl_4_+SKM (300 mg/kg)	29.43 ± 2.17^c^	58.23 ± 3.32^c^	0.48 ± 0.08^c^
SKM (150 mg/kg)	24.24 ± 1.32^a^	47.67 ± 2.31^d^	0.38 ± 0.09^e^
SKM (300 mg/kg)	23.78 ± 2.36^a^	48.03 ± 2.09^d^	0.39 ± 0.11^e^

SKM: *Seriphidium kurramense* methanol extract. Values expressed as mean ± SD (7). Means with different alphabet letters within the column indicate a significant difference (*p* < 0.05).

**Table 4:** Effects of SKM on the genotoxic parameters in CCl_4_-treated rats.

Treatment	Comet length (*μ*m)	Head length (*μ*m)	Tail length (*μ*m)	% DNA in the head	% DNA in the tail	Tail moment (*μ*m)
Control (normal)	62.14 ± 3.6^d^	55 ± 3.03^a^	7.3 ± 1.11^d^	91 ± 3.03^a^	9.01 ± 1.16^d^	31.51 ± 2.01^c^
DMSO+olive oil	62.23 ± 3.4^d^	55.5 ± 3.2^a^	7.5 ± 1.02^d^	90 ± 3.45^a^	9.86 ± 1.37^d^	31.42 ± 2.12^c^
CCl_4_ (1 mL/kg)	86.21 ± 4.2^a^	44 ± 3.33^c^	42 ± 2.11^a^	65 ± 2.12^d^	35 ± 2.56^a^	41.87 ± 2.07^a^
CCl_4_+silymarin	65.27 ± 3.1^c^	52.3 ± 3.1^b^	13.9 ± 1.23^c^	86 ± 3.16^b^	14.1 ± 1.9^c^	32.42 ± 2.01^c^
CCl_4_+SKM (150 mg/mg)	73.03 ± 3.3^b^	45 ± 3.42^c^	19 ± 2.45^b^	71.3 ± 3.1^c^	29.5 ± 2.3^b^	36.56 ± 2.02^b^
CCl_4_+SKM (300 mg/kg)	65.78 ± 3.3^c^	45.2 ± 2.3^c^	20.58 ± 1.4^b^	87.1 ± 3.2^b^	12.87 ± 1.1^c^	32.54 ± 2.04^c^
SKM (150 mg/kg)	61.62 ± 1.6^d^	54.3 ± 2.4a	7.31 ± 1.9d	91.2 ± 3.1a	9.78 ± 1.5d	30.50 ± 2.02c
SKM (300 mg/kg)	62.23 ± 1.3^d^	55.6 ± 2.2^a^	7.63 ± 1.01^d^	90.1 ± 4.1^a^	9.71 ± 1.13^d^	30.47 ± 2.04^c^

SKM: *Seriphidium kurramense* methanol extract. Values expressed as mean ± SD (7). Means with different alphabet letters within the column indicate a significant difference (*p* < 0.05).

**Table 5:** Distribution of animal groups (each containing 6 rats).

Group (control)	Given no treatment
Group 1 (control normal)	Normal healthy feeding
Group 2 DMSO+olive oil	Given 10% DMSO in olive oil orally (1 mL/kg rat body weight)
Group 3 (CCl_4_)	Given 30% CCl_4_ in olive oil i.p (1 mL/kg rat body weight)
Group 4 (silymirin+CCl_4_)	Given 30% CCl_4_ in olive oil i.p (1 mL/kg rat body weight)+silymarin (100 mg/mL in DMSO) orally
Group 5 (low dose+CCl_4_)	Given 30% CCl_4_ in olive oil i.p (1 mL/kg rat bodyweight)+*S. kurramense* methanol (SKM) (150 mg/kg rat body weight) orally
Group 6 (high dose+CCl_4_)	Given 30% CCl_4_ in olive oil i.p (1 mL/kg rat bodyweight)+*S. kurramense* methanol (SKM) (300 mg/kg rat body weight) orally
Group 7 (SKM)	Given only *S. kurramense* methanol (SKM) (150 mg/kg rat body weight) in DMSO orally
Group 8 (SKM)	Given only *S. kurramense* methanol (SKM) (300 mg/kg rat body weight) in DMSO orally

## Data Availability

The data used to support the findings of this study are available from the corresponding author upon request.

## Abbreviations

CCl_4_: Carbon tetrachloride
H_2_O_2_: Hydrogen peroxide
OOCCl_3_: Proxy trichloromethyl
CCl_3_: Trichloro-4 methyl
RO: Alkoxy radical
ROO: Peroxy radical
CAT: Catalase assay
POD: Peroxidase assay
SOD: Superoxide dismutase assay
GST: Glutathione S-transferase assay
GSH: Reduced glutathione assay
TBARS: Estimation of lipid peroxidation
PBS: Phosphate-buffered saline
LMPA: Low melting point agarose
NMA: Normal melting agarose
DMSO: Dimethyl sulfoxide
EDTA: Ethylenediaminetetraacetic acid
DNA: Deoxyribonucleic acid
CASP: Critical Appraisal Skills Programme
SKM: *Seriphidium kurramense* methanol extract
ALT: Alanine aminotransferase estimation
AST: Aspartate aminotransferase estimation
ALP: Alkaline phosphatase estimation.
